# Lower-Limb Muscle Contractile Properties, Explosive Power and the Subjective Response of Elite Soccer Players to the COVID-19 Lockdown

**DOI:** 10.3390/ijerph19010474

**Published:** 2022-01-01

**Authors:** Armin H. Paravlic, Bostjan Simunic, Sasa Pisot, Matej Kleva, Kaja Teraz, Matjaz Vogrin, Uros Marusic, Rado Pisot

**Affiliations:** 1 Faculty of Sport, Institute of Kinesiology, University of Ljubljana, 1000 Ljubljana, Slovenia; 2 Science and Research Centre Koper, Institute for Kinesiology Research, 6000 Koper, Slovenia; bostjan.simunic@zrs-kp.si (B.S.); sasa.pisot@zrs-kp.si (S.P.); matej.kleva@zrs-kp.si (M.K.); kaja.teraz@zrs-kp.si (K.T.); uros.marusic@zrs-kp.si (U.M.); rado.pisto@zrs-kp.si (R.P.); 3 Faculty of Sports Studies, Masaryk University, 625 00 Brno, Czech Republic; 4 Faculty of Medicine, Institute of Sports Medicine, University of Maribor, 2000 Maribor, Slovenia; matjazvogrin@hotmail.com; 5 Department of Health Science, Alma Mater Europaea—ECM, 2000 Maribor, Slovenia

**Keywords:** explosive power, CMJ, tensiomyography, male football, SJ, SARS-CoV-2

## Abstract

The present study examined the effects of the lockdown period on basic anthropometric measures, countermovement jumping performance, skeletal muscle contractile properties derived from tensiomyography (TMG), injury incidence, and self-assessed general well-being in elite soccer players. A total of 266 players were assessed before (PRE) and 32 players were reassessed 11 days after (POST) the COVID-19 period. Significant changes in the TMG parameters were observed POST compared to PRE: contraction time (Tc) increased from 6% to 50% in vastus lateralis [VL] (*p* = 0.009) and biceps femoris [BF] (*p* < 0.001), respectively; whereas radial displacement (Dm) increased for 19% in BF (*p* = 0.036) and 17% in VL (*p* < 0.001), respectively. Jumping performance remained unchanged from PRE to POST In addition, athletes rated the lockdown period as a positive event and felt psychologically better during the lockdown, primarily because they spent more time with family members and friends. Although there were no differences in any of the variables describing lower limb muscle power following the two-month lockdown, the altered contractile properties of the assessed muscles suggest suboptimal conditioning of the football players.

## 1. Introduction

The WHO recommended the adoption of numerous public health measures to halt the spread of the COVID-19 outbreak. These preventive measures mainly included lockdowns of cities, social distancing, and shutting down commercial services (e.g., trading, entertainment, sports and recreational facilities) to avoid social gatherings and diminish the chances of human-to-human virus transmission [1]. Consequently, in March–May 2020, all major sporting events were cancelled, while regular annual leagues were suspended until further notice. Countries worldwide began implementing the strategies mentioned above and as the ultimate countermeasure, governments ordered their citizens into self-isolation at home.

In most of the countries that were affected in the first wave of COVID-19 outbreak training schedules were compromised, especially in team sports and exercise with ball, resulting in training loads and muscular burden that could influence susceptibility for injuries and impair performance [2,3]. Athletes were confined and obliged to train at home no matter what type of sport they played. Slovenian soccer clubs provided their players with home-based training programs prescribed by their strength and conditioning coaches. However, given the strict nature of the lockdown and the level of physical fitness of the elite athletes, home-based training plans and programs could not simulate and replace sport-specific training stimuli and team practice to keep the soccer players in competitive shape. Therefore, during the COVID-19 lockdown period, athletes might be exposed to experiencing some level of deconditioning, despite regular home-based practice [4,5,6].

From surveying the general (also Slovenian) population, we found that the COVID-19 home lockdown had a negative effect on all physical activity intensity levels with 33% lower moderate and 33% lower vigorous physical activity, as well as 28.6% longer sitting time [7]. Furthermore, the COVID-19 home lockdown had a negative effect on mental wellbeing and emotional status with a 10–16.5% greater proportion of individuals experiencing psychosocial and emotional disorders. These psychosocial tolls were associated with unhealthy lifestyle behaviour, poor sleep quality, unhealthy diet [8,9,10] and significant decreases in social activity through family, friends/neighbours or entertainment [11]. However, not only the general public but also elite athletes experienced several new stressors during the COVID-19 pandemic lockdown. COVID-19 postponed all competitions and directly and indirectly limited their training programs—endangering their sporting season and career, isolating them socially, emotionally disrupting them, disconnecting them from typical healthy outlets and support networks, and losing concentration and motivation [12,13]. Although many subjective health consequences of the COVID-19 lockdown have been investigated, there are no studies reporting physical and performance consequences after the 1st wave (March–May 2020) of the COVID-19 lockdown.

Furthermore, others intended to raise awareness about possible scenarios and consequences for athletes’ health when elite soccer restarts [4,14]. Authors [5,10] urged the main decision-makers and stakeholders to focus on properly re-scheduling the competitions, making it possible for athletes to have an optimal period for regaining sport-specific conditioning and consequently to reduce the risk of eventual injuries [4,15,16].

The recent study [17] aimed to investigate COVID-19-induced quarantine effects on aerobic fitness and other parameters associated with the Yo-Yo intermittent test in professional soccer players. The authors [17] demonstrated less distance covered in both the relative and high-intensity efforts, lower maximum running speed, as well as acceleration and decelerations frequencies, whereas VO_2_max was not altered after quarantine. The latter results suggest that aerobic capacities could be preserved with a well-designed training plan, while neuromuscular performance associated with the Yo-Yo test is adversely affected. Rampinini et al., using a different methodological approach, namely comparing four different periods in two different seasons from before the COVID-19 lockdown, showed that negative alterations occurred for anaerobic performance, while positive effects were observed for the aerobic fitness of players. The authors concluded that aerobic fitness can be improved due to a greater volume of aerobic exercises conducted, while lower-limb strength training performed at home did not allow players to maintain the jumping ability of competitive periods [18].

To the authors’ knowledge, no study has examined neuromuscular performance in elite soccer players before and after the COVID-19 lockdown using tensiomyography (TMG). Since soccer players continued home-based training programs for approximately 8 weeks, we did not expect to observe anatomical changes in the skeletal muscle, rather functional ones (e.g., explosive power) [19]. Recent technological developments allow us to assess surrogate measures of muscle power i.e., contractile muscle properties, using TMG without fatiguing the muscle [20,21]. TMG is based on the non-invasive and selective assessment of skeletal muscle contractile properties using a displacement sensor placed on the skin over the individual muscle belly [22]. Several parameters of muscle contraction could be derived from TMG response, of which contraction time (Tc) and maximal radial displacement amplitude (Dm) have been shown to be the most reliable [20,21] and clinically relevant [23,24]. In recent years, it has been used extensively to measure muscle adaptations following disuse, training processes, and various rehabilitation protocols [23,24,25,26]. Furthermore, TMG was recently proposed as a sensitive method to observe hallmarks of muscle deterioration during the early phases of physical inactivity [27], which motivated us to apply the method for the first time in such a study.

Therefore, this study aimed to investigate the effects of the lockdown period on the basic anthropometrical measurements, muscle contractile properties, lower limb muscle power, the realization of training programs and changes in performance (technique, game tactics) and self-assessed general wellbeing and fitness level with an estimation of the impact of the lockdown on elite soccer players. We hypothesized that lower limb muscle power, TMG parameters and self-assessed general wellbeing will be negatively altered at POST, while an increased injury incidence should occur. This may help sports professionals better understand the consequences of lockdown on their athletes’ performance and better prepare them for future pandemics or waves of COVID-19 that can be expected [28].

## 2. Materials and Methods

### 2.1. Study Design

A longitudinal, observational cohort study was conducted to investigate the differences in anthropometry, lower limb TMG-derived muscle contractile parameters and muscle power from before to after the COVID-19 lockdown in elite soccer players. To address this issue, elite soccer players from the Slovenian Premier League were assessed before (PRE) and 11 days following (POST) the COVID-19 lockdown period. The PRE tests were performed in the framework of the national project (L5-8245), where the last measurements took place between 6 and 20 June 2019, and POST tests were additionally organized. Because of the specificity and exceptionality of the COVID-19 home lockdown situation in sports, we also assessed the athletes’ psychophysical and behavioural characteristics; however, during POST only.

### 2.2. Subjects

Two hundred and sixty-six elite soccer players (average age: 22.59 ± 3.91 years; body height 182.59 ± 6.44 cm; body mass 75.95 ± 6.63 kg) were assessed in the 2019/2020 soccer season. All the measurements took place between May and June of 2019. On 12 March 2020, the government of the Republic of Slovenia announced the epidemic due to the outbreak of the COVID-19 virus. Immediately, all gatherings were banned and all sports programs were suspended. Slovenian soccer clubs organized home-based training programs for their players until 8 May 2020. After that, players could return to their regular training, with all preventive measures still in place. Eleven days later, we were able to conduct the assessment in the players of two elite Slovenian soccer teams and managed to assess 32 players (average age: 24.97 ± 4.92 years; body height 181.53 ± 6.46 cm; body mass 76.19 ± 8.46 kg) from two elite soccer teams (ranked 2nd and 5th in the Slovenian first league at the time of the POST testing). Before proceeding with data collection, a brief meeting was held to explain the study protocol in detail where the written consent of each athlete was obtained as well. Only players with no injuries for at least 6 months prior to the assessment were eligible for the final analysis of performance-based measures. This study was approved by the Medical ethics commission at the Ministry of Health of the Republic of Slovenia, under the no. 0120-635/2017/4.

### 2.3. Procedures

All the procedures were carried out equally for PRE and POST (except the psychophysical and behavioural characteristics assessment) in the following order:

#### 2.3.1. Anthropometry

Body mass and height were measured using a stadiometer and scale (LIBELA ELSI, model SIGMA 5NP4, Celje, Slovenia) to the nearest 0.1 cm and 0.05 kg, respectively.

#### 2.3.2. Tensiomyography Assessment

The non-invasive TMG method was used to assess the contractile properties of three selected muscles: biceps femoris (BF) was performed prone at rest and with a knee angle set at 5° knee flexion; whereas the VL and vastus medialis (VM) were measured while supine at rest and with a knee angle set at 30° knee flexion. Foam pads were used for leg support. The oscillations of the belly muscle in response to an electrically induced isometric twitch were recorded at the skin surface using a sensitive digital displacement sensor (TMG-BMC, Ljubljana, Slovenia). The sensor was set perpendicular to the skin’s normal plane above the belly muscle: at the midpoint of the line between the fibula head and the ischial tuberosity on the BF; at 30% of the femur length above the patella on the VL and four fingerbreadths on the VM, proximal to the superior-medial angle of the patella. The reliability of these measuring positions is below 3% [29]. The rounded (5-cm diameter) self-adhesive cathode and anode (Axelgaard, Aarhus, Denmark) were set 5 cm distally and 5 cm proximally to the measuring point on all muscles assessed [30]. Electrical stimulation was applied through a TMG-100 System electro stimulator (TMG-BMC d.o.o.) with a pulse width of 1 ms and an initial amplitude of 30 mA. During each testing session, the amplitude was progressively increased by 20 mA increments until there was no further increase in the amplitude of the TMG response (Dm). Rest periods between stimuli of 10 s were given between each stimulus to minimize the effects of fatigue and potentiation. More detailed testing procedures were previously described elsewhere [20,31]. From two maximal twitch responses, the TMG parameters were calculated as follows: Dm—maximal amplitude; delay time (Td) as the time from the electrical impulse to 10% of the Dm; contraction time (Tc) as the time from 10% to 90% of Dm; sustain time (Ts) as the time from 50% to 50% of the Dm; and half-relaxation time (Tr) as the time from 90% to 50% of the Dm.

#### 2.3.3. Lower Limb Muscle Power

The lower limb muscle power was assessed using vertical jump tests, where a KISTLER Quattro jump (Type 9290CD; 920 cm × 920 cm × 125 cm, linearity ± 0.5%, range 0–10 kN: sampling rate of 1000 Hz; Kistler Instrument Corp., Winterthur, Switzerland) force platform was used. The vertical jump performance was assessed following the standardized warm-up routine consisting of 6-min of stepping-up and 5 min of whole-body dynamic stretching. After the warm-up routine, 2–3 warm-up countermovement jumps (CMJ) and squat jumps (SJ) without hands were executed, followed by actual maximal jump trials. Briefly, the players stood with their hands on their hips on the force platform. Starting from the half-squat static position (knees at approximately 90°) (SJ test) or the free countermovement position (CMJ test), the subjects were told to jump as high as possible in each of the three attempts required. The subjects were instructed and verbally encouraged to maximize their jump height, with the highest jump being taken for further analysis. Through a visual inspection, take-off and landing were standardized to the same spot and the players were required to perform a triple extension (i.e., full hip, knee, and ankle extension) during the flight phase, with the same full extension on landing. The players were required to achieve a proper jump technique, otherwise they repeated the jumping trial after a short rest period until a valid attempt was achieved. Finally, the jump (both CMJ and SJ) with the maximal achieved height calculated from take of velocity was taken for further analysis. Additionally, the eccentric utilization ratio (EUR) and reactive strength index modified (RSImod) were reported. The RSImod was calculated as the CMJ jump height divided by the takeoff time [32], while the EUR was calculated as follows: [(CMJ height − SJ height)/SJ height] × 100 [33].

#### 2.3.4. The Lockdown Impact Characteristics Questionnaire Survey

The questionnaire was created to gather additional information from the athletes on the estimation of their daily activities, as well as physical and psychological wellbeing and behavioural changes during the COVID-19 lockdown (Questionnaire S1).

The questionnaire was constructed by a panel of researcher on the basis of research question which were selected by them. In accordance with the topic of injury being studied, the researchers (SP, AP, BŠ, MK, RP) collected data of interest in addition to the data obtained through measurements executed within the protocol of the Qsport project. Furthermore, a series of potential questions were prepared, which were reviewed by the aforementioned researchers and checked for understanding by completing a test version of the questionnaire. The questionnaire was also pilot tested on a number of athletes. The questionnaire interview was conducted between a researcher (SP) and an athlete due to the parallel measurements and in a way that was easier for the athlete. At the same time this procedure prevented the possibility of questions being omitted or misunderstood. The questionnaire has been prepared in an Excel document so that researcher (SP) can directly insert athletes’ answers.

The questionnaire consisted of eight questions mainly focused on the self-assessment of home-based training that the soccer club provided to their athletes during the lockdown period.

In the questions (Q2–Q5) the athletes had to estimate the percentage of plan realization and the possible adaptation of the plan (due to injuries and/or rehabilitation processes). If 90–100% of the plan was not implemented, they provided a reasonable explanation. A subjective assessment of the training program was based on the level of difficulty/intensity (1—easy, not difficult at all, 2—not intense enough or difficult enough, 3—adequate, 4—a little too difficult or too intense, 5—much too intense or too difficult).

In addition, the athletes were asked to assess the extent to which they observed changes during the COVID-19 lockdown (Q6—Q6e) in:

(a) Their body mass. A 5-point Likert-type scale was used, with the values interpreted as follows: 1—a decrease of more than 2 kg; 2—a decrease from 0.5 to 2 kg), 3—stayed the same, 4—an increase from 0.5 kg to 2 kg, 5—an increase of more than 2 kg;
(b) And general well-being; physical fitness, technique and game tactics. Again, the 5-point Likert scale was used, with the values interpreted as follows: 1—much worse, 2—slightly worse, 3—the same, 4—better, 5—much better).

For the last questions (Q7), we adopted the “The Life Events Survey for Collegiate Athletes—LESCA” in which the athletes were asked to indicate what kind of impact the COVID-19 home lockdown had on their live events (−4—extremely negative, −3—negative, −2—moderately negative, −1—somewhat negative, 1—somewhat positive, 2—moderately positive, 3—positive, 4—extremely positive) [34]. Athletes were asked (Q7): How did this event—the state of restrictive measures- affected them and additionality with Q7a if any other event (e.g. illness in the family, death of a loved one, birth, marriage, divorce from partner, change in sleeping habits, different eating other habits…) occurred during the lockdown of sport and had an impact on them, where they need to name the event and estimate (in the scale form −4 to 4) the impact of the event.

### 2.4. Statistical Analysis

All the data is presented as mean ± SD. All statistical analyses were performed using the SPSS statistical software (version 19.0, IBM Inc., Chicago, IL, USA). Descriptive statistics was used to summarize the demographic characteristics and outcome measures. Normality was confirmed by visual inspection and using the Shapiro-Wilk test, while the homogeneity of variances was tested using Levene’s test for all dependent variables. Student’s paired sample t-test was used to investigate the differences between PRE and POST. Statistical significance was accepted at *p* < 0.05. Additionally, the magnitude of the changes from PRE to POST were presented as raw mean differences (RMD) and the percent of change. When significant differences were found, a Cohen d was estimated.

The frequencies and means with SD were used to represent the questionnaire data. The statistical analyses of the questionnaire were performed using Microsoft Excel^®^ for Microsoft 365MSO (16.0.13628.20128).

## 3. Results

This section was divided by subheadings. It was intended to provide a concise and precise description of the experimental results, their interpretation, as well as the experimental conclusions that can be drawn.

### 3.1. Training Adherence and Body Mass during the COVID-19 Lockdown Period

Although 266 players (all 10 Slovenian soccer teams) were examined at PRE, a subsample of 32 players (from only two teams) were measured at POST (during the lockdown period, 81% of the players reported being able to train at 90–100% of the given training plan and program, 19% reported being able to train at 75–80%, of whom four players adopted the training plan as a part of the rehabilitation process due to a previous injury. In addition to injuries, the main reasons for not being able to train at 100% of the strength and conditioning programme, given by their coach, were reported as follows: 40% reported training monotony (e.g., no soccer-specific exercises/drills with the ball) and 12.5% reported a lack of motivation, while others reported a lack of conditions for adequate training. The training programme was subjectively rated by players as optimal to difficult to manage and achieved an average score of 3.7 (0.7) out of 5.

The players estimated the change in body mass as −0.1 (0.8) points on average, with 60% of them reporting no change during the period, nine players (28%) reporting body mass losses of 0.5 to 2 kg and four of them (12.5%) reporting body mass gains of 0.5 to 2 kg.

### 3.2. Skeletal Muscle Contractile Properties by Tensiomyography

**Figure 1:**
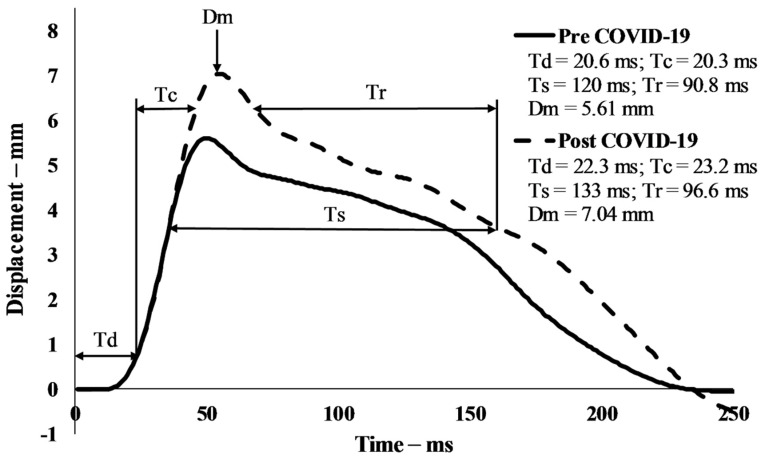
Tensiomyographic responses of the vastus lateralis muscle from before (solid line) and after (dashed line) the two-month lockdown period, an example of a single representative subject. Td—delay time; Tc—contraction time; Ts—sustain time; Tr—half-relaxation time; Dm—amplitude.

When the POST values were compared to the PRE-values, the TMG parameters had changed in all the observed muscles (Table 1). Td increased consistently from 9.4% (VM) and 10.8% (VL) to 19.6% (BF), all *p* < 0.001. The same goes for Tc, which increased from 5.6% (VL; *p* = 0.009) to 50.2% (BF; *p* < 0.001), while it decreased in VM (4.1%; *p* = 0.012). Ts only increased in VL (62.4%; *p* = 0.032) while Tr only decreased in VM (47.6%; 0.001). Dm increased in BF (18.8%; *p* = 0.036) and VL (16.8%; *p* < 0.001). An example of a single representative subject for tensiomyographic responses of the vastus lateralis muscle from before and after the two-month lockdown period was presented in Figure 1.

### 3.3. Lower Limb Muscle Power

Compared to PRE, there were no differences in either variable of lower limb muscle power assessment (all *p* > 0.072) (Table 2).

### 3.4. Subjective Ratings of Performance Changes and Stress during COVID-19 Lockdown Period

When self-assessing the changes due to the COVID-19 home lockdown in terms of the athletes’ general well-being, physical fitness, technique and game tactics, half of the athletes reported no changes in terms of general well-being, with an average of 3.2 (0.8) points, with 15% of the athletes feeling worse or much worse and almost 35% feeling better (Table 3). Forty percent of the athletes estimated a negative change in physical fitness (worse or much worse) and in contrast, almost 44% estimated a positive change (better or much better), while 16% had no change, i.e., an average of 3.1 (1.1) points. Technique (2.7, (0.6) points on average) and game tactics (2.9 (0.6) points on average) were rated as not changed or remaining the same (60% in technique and 63% in-game tactics). For one-third of the athletes, the technique was rated as slightly worsened and only two of them reported an improvement. For in-game tactics, only 25% of the athletes reported a slight deterioration while as many as 12.5% reported an improvement.

In general, 75% of athletes rated the COVID-19 lockdown situation as a positive “life event” (12.5% even extremely positive), with 1.0 (1.9) points on average, mainly because they had more time to spend with their family members and had time to work on themselves; in contrast, 25% of them rated it as somewhat or moderately negative when they were separated from family and social life in the COVID-19 lockdown period (Table 4).

## 4. Discussion

This study investigated the effects of the COVID-19 first wave lockdown period on the TMG-derived lower limb skeletal muscle contractile properties, jumping power home-based training adherence and psychosocial response of elite first league soccer players. The main findings were that: (1) TMG parameters changed in all muscles measured: (2) the jumping performance remained unaltered; (3) the athletes estimate the period of lockdown as a positive event and felt psychologically better during the lockdown, primarily because of more time spent with family members and friends.

### 4.1. Impact of COVID-19 Lockdown on Lower-Limb Muscle Contractile Properties Assessed by Tensiomyography

To our knowledge, this is the first study showing the response of TMG derived parameters and lower limb muscle power performance after an in-season break caused by the lockdown due to the COVID-19 outbreak. TMG has been extensively used to measure adaptations of skeletal muscle contractile properties in different settings [23,24,25,27,35]. From the TMG response, several parameters of muscle contraction could be derived, of which Tc and Dm were found to be the most reliable [20] and clinically relevant [23,24,27]. Following eight weeks of lower limb plyometric training, Zubac and Šimunič [35] reported decreased Dm (ranging from 6% to 27%), Tc (ranging from 8% to 27%), and the estimated VL MHC-I proportion (8%), which was paralleled by an increase in the countermovement jump height of 12%. In contrast, the results of the present study showed a significant increase in the Dm of weight-bearing VL (16.8%) and non-weight-bearing BF (18.8%) muscles but not in VM. An increase in Dm could be explained by muscle tone loss, fluid redistribution, muscle architecture change [27], or even muscle atrophy [23], which are regularly found in young adults after prolonged physical inactivity. In one of the first studies aiming to investigate the relationship between muscle thickness and Dm, Pišot et al. [23] reported that an increase in Dm was paralleled and correlated with a decrease in muscle thickness following 35 days of bed rest [27,36]. However, Šimunič et al. [27] found increased Dm much sooner. They were evident with ultrasound inspection during the 35-day bed rest indicating the high sensitivity of TMG for the detection of early atrophy processes. Another interesting finding is that participants with stiffer muscles, with a lower Dm than usually found in athletes and after intense exercise [35], at baseline were prone to greater muscle atrophy following muscle disuse [23], which may be the case with well-trained individuals following insufficient training stimuli [19].

The two months of lockdown induced an increase in the VL Tc (5.6%); however, a much greater increase in Tc was observed in BF (50.2%). A similar, disproportional change was also found after 35 days of bed rest, while no change was reported for knee extensors (VM), but a 39.2% increase was found in BF, and this increase in BF Tc was irreversible for at least 30 days of the recovery that followed [27]. The observed difference in response between muscles could be partly explained by the higher proportion of fast-twitch fibres in the knee extensors that are more resilient to inactivity-induced muscle atrophy [37]. It is also important to be aware that knee flexors, which are antigravitational muscles, experience higher loadings, which both muscles undergo during normal daily activity such as body mass support and propulsion during locomotor activities. Whereas BF is not a weight-bearing muscle and undergoes much lower loading during daily activities [38] and therefore is subject to a higher degree of deterioration when limiting daily activities during the COVID-19 lockdown restrictions. Moreover, the higher plasticity of BF corresponds to those alterations observed in different populations [27]. Šimunič et al. [27] compared the BF Tc data of different populations, from children and athletes to older adults. When the BF Tc of young men (aged 28 years) was compared to a sedentary older group of adults (aged 65 years), the greatest difference of 56.5% was observed.

While the aforementioned studies, relying on bed rest investigations, represent an extreme type of physical deconditioning, they do reveal possible mechanisms for the changes observed in the current investigation. Although the athletes assessed in the present study were provided with an individualized training plan, the lack of adequate equipment, sports facilities and sport-specific locomotor activities resulted in some type of deconditioning i.e., reduced physical activity and sport-specific exercise. The 18.8% and 16.8% increase in the Dm of the BF and VL muscles provides additional support for the thesis that elite players with higher levels of trainability may impair their muscle structure during a prolonged period of reduced training.

### 4.2. Impact of COVID-19 Lockdown on Lower-Limb Muscle Power

The literature postulates that both short-(less than 4 weeks in duration) and long-term deconditioning (more than 4 weeks in duration) may cause significant alterations to cardiorespiratory fitness, metabolic profile, muscular characteristics and consequently to soccer-specific performance [19,39,40,41,42,43]. For example, Joo [43] showed that two weeks of detraining can markedly impair performances in the Yo-Yo and repeated sprints tests in professional soccer players. Similarly, CMJ and SJ performance can be expected to decline by between 2.6% and 4.7%, as well as the sprinting time in both 10 m (2.9%) and 20 m (1.4%) tests following four and/or six weeks of detraining in competitive soccer players, showing the length of exposure dependence [39,41]. Moreover, one recent study [17] aimed to investigate COVID-19-induced quarantine effects on aerobic fitness and the Yo-Yo intermittent test associated measurements in professional soccer players. Authors [17] demonstrated less distance covered in both the relative and high-intensity efforts, lower maximum running speed, as well as acceleration and decelerations frequencies, whereas Vo2max was not altered after quarantine. The latter results suggest that aerobic capacities could be preserved with a well-designed training plan, while neuromuscular performance associated with the Yo-Yo test is adversely affected. Contrary to the literature, we did not reveal any changes in neuromuscular performance by utilizing vertical jump performance tests. These results can be explained by different study designs, the trainability level of the athletes and the measurements assessed. Moreover, given the specificity of the jumping task to common soccer-related movement patterns, it could be speculated that task-dependent motor control/muscle memory kept the jumping performance unaltered [44,45].

### 4.3. Subjective Ratings of Performance Changes and Stress during COVID-19 Lockdown Period

As the global lockdown response to the COVID-19 pandemic implies social isolation or home confinement, the new daily routine of “stay home” also impacts athletes. Luckily in Slovenia, the first wave did not impose large-scale infection and death as in the second wave [46], so the lockdown of sports events (soccer national league) lasted less than two months (from 16 March to 9 May 2021) when the athletes could start training under restrictive conditions imposed by the government. The two months of adapted conditions, with no matches and a flexible training plan probably created a space for reimagining ourselves and our preferred futures, probably reflected the high number of athletes that estimated the COVID-19 lockdown experience as a positive “life event” since players had more time to spend with their family and to work on themselves. Consequently, a quarter of the players showed a negative impact from being separated from their families during the lockdown and perceived this time as a negative experience of a lack of social contact, indicating the importance of emotional balance provided by family or partner [47]. The current study consisted of elite first league male soccer players from two top-ranked Slovenian teams and the present results should not be generalized to recreationally trained or untrained populations, or female subjects.

A limitation of the present study is that it was retrospectively planned, whereas the baseline assessment was not conducted immediately before the lockdown period, but 11 months before. Therefore, the large variations in the individual players’ trainability level may have occurred. It should be noted, however, that during the screening process, subjects were excluded if they had an individualized training plan because of injury or illness.

## 5. Conclusions

In conclusion, the TMG-derived skeletal muscle contractile properties (Dm and Tc) changed in all the muscles measured. Even though there were no differences in any of the variables used to describe the lower limb muscle power following the two-month lockdown period, the altered contractile properties of the assessed muscles suggest the suboptimal conditioning of football players leading to increased injury incidence in the two months after the easing of the COVID-19 restrictions.

## 6. Perspectives

The results of the present study highlight the relevance of monitoring individual skeletal muscle contractile properties rather than using only physical performance tests. Therefore, it is important for coaches and athletes to incorporate techniques for detailed skeletal muscle testing into their general training protocols to adapt the training process and potentially reduce the occurrence of musculoskeletal injuries. They should also be aware of the importance of a balanced psychological environment for elite soccer players in times of similar extraordinary circumstances that we have witnessed during the COVID-19 pandemics.

## Figures and Tables

**Table 1:** Comparison of the tensiomyographic characteristics of dominant leg muscles at baseline, from pre-lockdown, (PRE) to post-COVID-19 lockdown (POST).

Muscle	Parameter	PRE	POST	RMD	*p* Value (Cohen’s d)
BF	Td	23.76 ± 2.94	28.42 ± 4.01	4.65	**<0.001 (1.58)**
	Tc	28.25 ± 8.91	42.42 ± 14.71	14.18	**<0.001 (1.59)**
	Ts	202.7 ± 40.06	191.2 ± 25.42	−11.49	0.078
	Tr	51.29 ± 29.80	48.33 ± 16.81	−2.95	0.641
	Dm	6.57 ± 2.73	7.80 ± 2.44	1.23	**0.036 (0.45)**
VL	Td	20.63 ± 1.57	22.86 ± 1.23	2.23	**<0.001 (1.42)**
	Tc	19.91 ± 2.24	21.02 ± 1.93	1.11	**0.009 (0.50)**
	Ts	75.16 ± 110.5	122.0 ± 33.95	46.87	**0.032 (0.42)**
	Tr	68.95 ± 105.0	74.80 ± 29.50	5.85	0.766
	Dm	5.30 ± 1.43	6.19 ± 1.16	0.89	**<0.001 (0.62)**
VM	Td	21.21 ± 1.84	23.21 ± 1.68	2.0	**<0.001 (1.09)**
	Tc	22.92 ± 2.38	21.99 ± 1.99	−0.93	**0.012 (0.39)**
	Ts	209.0 ± 34.11	197.0 ± 25.97	−12.05	0.080
	Tr	104.3 ± 68.82	54.70 ± 42.95	−49.61	**0.001 (0.72)**
	Dm	7.65 ± 1.32	7.34 ± 1.40	−0.31	0.226

VL—vastus lateralis; VM—vastus medialis; BF—biceps femoris; Bolded value—significant difference; RMD—raw mean difference; Cohen d—Effect size.

**Table 2:** Comparison of anthropometric and selected sport-related physical performance measures at baseline, from pre-lockdown, (PRE) to post-COVID-19 lockdown (POST).

	PRE	POST	RMD	*p* Value (Cohen’s d)
Age (years)	24.97 ± 4.92	25.56 ± 4.93	0.59	**<0.001 (0.12)**
Body height (cm)	181.53 ± 6.46	181.53 ± 6.46	0.0	
Body mass (kg)	76.19 ± 8.46	77.75 ± 8.13	1.56	**0.003 (0.18)**
Body mass index (kg/m^2^)	23.07 ± 1.62	23.55 ± 1.51	0.48	**0.003 (0.30)**
CMJ height (cm)	38.27 ± 4.85	38.55 ± 5.83	0.28	0.599
SJ height (cm)	35.40 ± 4.30	36.22 ± 5.21	0.82	0.380
EUR (%)	8.59 ± 10.91	6.61 ± 5.94	−1.97	0.379
Vertical take-off velocity (m/s)	2.53 ± 0.74	2.74 ± 0.21	0.2	0.145
Mean power (W)	2437.5 ± 298.1	2434.5 ± 269.1	3.0	0.942
Take off time	0.76 ± 0.08	0.73 ± 0.07	0.03	0.072
Reactive strength index (CMJ)	0.509 ± 0.067	0.537 ± 0.106	0.03	0.131

CMJ—Countermovement jump; SJ—Squat jump; EUR—eccentric utilization ratio; Bolded value—significant difference; RMD—raw mean difference; Cohen d—Effect size.

**Table 3:** The athletes’ observed changes during the COVID-19 lockdown.

	General Well-Being	Physical Fitness	Technique	Game Tactics
Much worse	3.1%	3.1%	0.0%	0.0%
Slightly worse	12.5%	37.5%	34.4%	25.0%
The same	50.0%	15.6%	59.4%	62.5%
Better	31.3%	37.5%	6.3%	12.5%
Much better	3.1%	6.3%	0.0%	0.0%

**Table 4:** Impact of the COVID-19 home lockdown on athletes’ lives.

Impact of COVID-19 Home Lockdown	Frequency/%
Extremely positive	12.5
Positive	15.6
Moderate positive	15.6
Somewhat positive	31.3
Somewhat negative	15.6
Moderate negative	9.4
Negative	0.0
Extremely negative	0.0

## Data Availability

All data generated are available within the present manuscript.

## Supplementary Materials

The following supporting information can be downloaded at: www.mdpi.com/article/10.3390/ijerph19010474/s1, Questionnaire S1: The Lockdown Impact Characteristics Questionnaire Survey.
