# Supplementary material for: Lower-Limb Muscle Contractile Properties, Explosive Power and the Subjective Response of Elite Soccer Players to the COVID-19 Lockdown
Source: Int J Environ Res Public Health. 2022 Jan 1;19(1):474. doi: 10.3390/ijerph19010474 (PMC8744705; doi:10.3390/ijerph19010474)
Supplement: Supplementary file 1 [file ijerph-19-00474-s001.zip › ijerph-1508949-supplementary.pdf]

Description of the Questionnaire – post CQOVID-19 measurement of football players

The questionnaire was crated within the purpose to gathered the additional information from the athletes (football players of NK MURA and NK Maribor, national 1st league) regarding estimation of their basic actions, physical and psychological state and behaviour of athletes did or feel during the COVID-19 pandemic state.

The questionnaire was constructed by a panel of researcher on the basis of research question which were selected by them. In accordance with the topic of injury being studied, the researchers (SP, AP, BŠ, MK, RP) collected data of interest in addition to the data obtained through measurements executed within the protocol of the Qsport project. Furthermore, a series of potential questions were prepared, which were reviewed by the aforementioned researchers and checked for understanding by completing a test version of the questionnaire. The questionnaire was also pilot tested on a number of athletes. The questionnaire interview was conducted between a researcher (SP) and an athlete due to the parallel measurements and in a way that was easier for the athlete. At the same time this procedure prevented the possibility of questions being omitted or misunderstood. The questionnaire has been prepared in an Excel document so that researcher (SP) can directly insert athletes answers.

The questionnaire consisted of 8 questions and encompass questions regarding the time the sports club provide them the plan of individual training.

In the questions (Q2- Q5) athletes had to estimate the percentage of the plan realisation the possible adaptation of the plan because of injurie, rehabilitation process etc. and if the plan was not realised in 90-100%, they need to name also the reason for that. The estimation of training program (plan) was provided on the 1 to 5 point scale of level of difficulty/intensity (1-to easy, not difficult at all, 2- to little intensive or difficult, 3– appropriate, 4- little to difficult or to intensive, 5- far to intensive or difficult).

In additional self-report scale (Q6 – Q6e) athletes need to estimate to what extend they observe changes during the COVID-19 sports quarantine in:

a) body mass on the scale of 1-5) where mean:1- decreased more than 2 kg; 2 - decreased from 0,5 to 2kg), 3-stays the same, 4- increased from 0,5 kg to 2kg, 5-increased more than 2 kg.

b) and general well-being; physical fitness, technique, game tactics on the scale from 1-5 (1- much worse, 2- slightly worse,3- the same, 4-better, 5-much better).

Last question (Q7) referred to the estimation of general impact of COVID-19 pandemic experience on the individual athlete. For this purpose, we adopted The Life Events Survey for Collegiate Athlete- LESCA **(**Petrie, 1992;) question" to indicate what kind of an effect this event had on your life when the event occurred" on the scale: extremely negative =-4; negative= - 3, moderate negative = - 2; somewhat negative = -1; somewhat positive = + 1; moderate positive + 2; positive == +3; extremely positive =+4.

Athletes were asked (Q7): How did this event - the state of restrictive measures- affected them and additionality with Q7a if any other event (e.g illness in the family, death of a loved one, birth, marriage, divorce from partner, change in sleeping habits, different eating other habits…) occurred during the lockdown of sport and had an impact on them, where they need to name the event and estimate (in the scale form -4 to 4) the impact of the event.

Reference:

Trent A. Petrie PhD (1992) Psychosocial Antecedents of Athletic Injury: The Effects of Life Stress andSocial Support on Female Collegiate Gymnasts, Behavioral Medicine, 18:3, 127-138, DOI: 10.1080/08964289.1992.9936963
